# Analysis of the interaction network relationship between drugs using a graph neural network

**DOI:** 10.3389/fphar.2026.1686243

**Published:** 2026-06-12

**Authors:** Zhongyi Chai, Jing Wang, Huili Du

**Affiliations:** 1 Department of Cardiology, The First Affiliated Hospital of Zhengzhou University, Zhengzhou, China; 2 Xinxiang Central Hospital, The Fourth Clinical College of Xinxiang Medical University, Xinxiang, China

**Keywords:** biomedical informatics, graph attention networks, knowledge graph, multi-relational learning, uncertainty modeling

## Abstract

**Introduction:**

The ever-increasing complexity of biochemical systems, alongside the rapid growth of pharmaceutical and biomedical data, underscores the urgent need for intelligent, scalable, and interpretable computational models. These models must be capable of supporting next-generation decision-support systems and driving knowledge discovery in the realm of computational science. Traditional approaches to relational biomedical modeling, however, often struggle to accurately capture intricate multi-relational dependencies and typically lack robustness in sparse or incomplete interaction domains. To address these pressing limitations, we present a novel, biologically grounded graph-based learning framework designed to overcome such challenges.

**Methods:**

Our approach comprises a two-tiered system: PHARMNet, a multi-relational graph neural network (GNN) equipped with memory-augmented attention mechanisms, and INTERACT-SCOPE, an advanced, context-aware optimization strategy that leverages structured biomedical ontologies and domain knowledge. PHARMNet employs relation-specific graph convolutions and semantic embedding alignment to effectively model latent relational dependencies in biochemical and pharmacological datasets. In parallel, INTERACT-SCOPE improves predictive generalization and stability by incorporating ontology-guided constraints, estimating epistemic uncertainty, and applying adaptive graph regularization techniques tailored to biomedical structures.

**Results and Discussion:**

Through rigorous experimental evaluations across a variety of pharmacological interaction categories, our framework consistently achieves state-of-the-art (SOTA) predictive performance, enhanced model interpretability, and notable robustness—especially in low-data or high-noise scenarios. These outcomes strongly align with the journal’s mission to promote innovative and knowledge-driven advances in software engineering, artificial intelligence, and biomedical informatics. Ultimately, our article illustrates the synergistic integration of computational intelligence, domain-informed graph representation learning, and scalable modeling, contributing a powerful and interpretable solution to real-world challenges in healthcare informatics and biomedical discovery. Experimental results demonstrate that MGTNSyn outperforms existing methods, achieving an AUC of 0.873 and an F1-score of 0.831 on drug–drug interaction (DDI) benchmark datasets.

## 1 Introduction

Understanding the interaction network relationships between drugs is crucial for accelerating drug development, improving pharmacovigilance, and identifying potential drug–drug interactions (DDIs). As the pharmaceutical landscape rapidly evolves, deciphering how compounds interact within intricate biological systems becomes increasingly vital (Hao et al., 2025). Traditional drug interaction studies have typically depended on labor-intensive laboratory experiments or manual curation of databases, both of which are insufficiently scalable for the exponential growth of biomedical data. Given the serious health risks posed by undetected DDIs, there is an urgent need for intelligent, automated models that can predict interactions using structural, pharmacological, and molecular information (Singha et al., 2020).

Recent advances in artificial intelligence (AI) and deep learning have significantly impacted the pharmaceutical sciences, offering new avenues for drug discovery, toxicity prediction, and treatment optimization. Neural networks, in particular, are increasingly being employed to model complex nonlinear relationships between chemical, biological, and clinical features, enabling the prediction of pharmacokinetic and pharmacodynamic behaviors of drugs. Their ability to learn directly from high-dimensional and heterogeneous data has made them suitable for various applications, including molecular property prediction, target identification, and personalized medicine. As highlighted in recent studies, AI-based modeling is proving to be a transformative tool in accelerating pharmaceutical research and improving drug safety profiles (Suriyaamporn et al., 2024; Vora et al., 2023). Building on this foundation, in this study, we focus on the use of graph neural networks (GNNs), which are especially well-suited for modeling molecular structures and interaction networks due to their graph-based architecture. In recent years, the adoption of computational approaches has become more prevalent, enabling researchers to manage complex datasets with greater efficiency and extract actionable insights that would otherwise remain hidden. Among the emerging solutions, network-based models—especially those leveraging graph structures to represent drugs and their interrelations—have shown significant promise. These models provide a flexible framework to capture the rich and diverse relationships between compounds, enabling more comprehensive interaction mapping. GNNs, in particular, have proven effective in learning from non-Euclidean data structures, capturing both localized node features and broader topological patterns (Khatir et al., 2022). By integrating information across multiple layers of the network, GNNs enhance the model’s capacity to generalize from known interactions and infer novel interactions. These capabilities not only improve predictive accuracy but also support the discovery of previously unknown interactions that traditional analytical methods might overlook (Zhao et al., 2010). As biomedical data continue to grow in complexity and volume, GNN-based models stand out as a scalable, data-driven solution for addressing the challenges of modern drug interaction analysis. Early computational efforts focused on integrating curated domain knowledge with structured rules to infer potential DDIs (Percha and Altman, 2013). These approaches typically relied on well-established pharmacological classifications, hierarchical taxonomies, and adverse event reporting systems to construct interaction networks (Chautard et al., 2009). For example, drugs were categorized based on their therapeutic class, chemical structure, or target mechanisms, and interaction rules were manually encoded to simulate the logic of expert reasoning (Ruffner et al., 2007). By mapping these relationships into structured frameworks, researchers were able to generate interpretable models that aligned with known clinical guidelines and supported regulatory decision-making. Such systems often demonstrated high precision when applied to known interactions, making them valuable in safety-critical applications where transparency and explainability were paramount. Moreover, their ability to reflect domain knowledge made them particularly suitable for integration with electronic health records, drug labeling systems, and pharmacovigilance databases (Lin et al., 2007). However, despite these strengths, the effectiveness of rule-based systems was constrained by several inherent limitations. Most notably, they depended heavily on predefined ontologies and manually curated knowledge bases, which required continuous updates to remain relevant (Hase et al., 2009). This made them inflexible and poorly suited for detecting interactions involving newly approved or investigational compounds, for which prior information might be limited or unavailable. Their static nature also hindered their ability to adapt to emerging scientific discoveries or incorporate real-time data from rapidly expanding biomedical repositories (Fan et al., 2021). Furthermore, these models struggled to reconcile heterogeneous sources of information—such as genetic variation, metabolic pathways, and polypharmacy patterns—which are increasingly important for understanding the nuanced mechanisms behind DDIs. As a result, their capacity to generalize beyond known data and uncover novel interactions was limited. These challenges highlighted the need for more dynamic, flexible, and data-responsive modeling paradigms that could evolve alongside biomedical knowledge and better capture the complex, interconnected nature of drug interaction networks in real-world settings (Yang et al., 2019).

As biomedical datasets became increasingly richer and more multifaceted, encompassing a wide array of chemical, biological, and clinical information, researchers began to transition from static rule-based systems to more flexible, statistical modeling approaches. These methods sought to uncover latent interaction patterns by leveraging numerical representations of drug characteristics, thereby enabling automated learning from large-scale data collections. Typically, these approaches extracted descriptors such as molecular fingerprints, chemical substructures, protein-binding profiles, pharmacokinetic properties, and known adverse effects (Li et al., 2017). With these features as inputs, a variety of classification algorithms—ranging from support vector machines and logistic regression to random forests and gradient boosting—were trained to predict potential DDIs (Takeda et al., 2017). Ensemble techniques and margin-based classifiers, in particular, offered improved scalability and robustness, demonstrating the ability to outperform traditional models in various benchmark tasks. These statistical models marked a significant advancement because they allowed data-driven inference and supported predictions for unseen drug pairs. However, their performance was critically dependent on the quality, relevance, and completeness of manually engineered features. Feature engineering required substantial domain expertise to select the most informative attributes and design transformation strategies, introducing potential biases and inconsistencies across datasets. Moreover, these handcrafted features often failed to capture the intricate relational and contextual dependencies between drugs that are essential for modeling complex biochemical interactions (Letychevskyi et al., 2023). In scenarios characterized by sparse, noisy, or high-dimensional data, these models frequently encountered difficulties with generalization and robustness, leading to reduced reliability in real-world applications. Because the majority of conventional classifiers treated drug pairs as independent observations, they overlooked the global structure and topological context of the interaction network, limiting their ability to detect higher-order relationships or infer mechanisms beyond pairwise associations. These limitations underscore the need for more expressive modeling frameworks capable of automatically learning rich, structured representations while integrating multiple layers of biomedical information. This has sparked a growing interest in neural architectures—particularly those designed to handle graph-structured data—offering a pathway toward more adaptive and biologically grounded solutions for DDI prediction (Goodman and Walsh, 2001).

In response to the limitations of earlier approaches, recent advancements have increasingly turned to deep learning frameworks capable of capturing both the intrinsic properties of drugs and the intricate structure of their interactions. Among these, GNNs have emerged as particularly promising due to their ability to model drug interaction networks in a relational and context-aware manner. In GNN-based models, drugs are represented as nodes, whereas known or potential interactions form the edges of a graph. This representation enables the model to learn not only from individual drug features—such as molecular structure, target proteins, and known side effects—but also from the topology of the network itself. Through iterative message-passing and information aggregation mechanisms, GNNs dynamically update node embeddings to reflect both local and global relational contexts, thereby uncovering high-order dependencies and latent semantic patterns within the interaction network (Dara et al., 2024). Various GNN architectures have been proposed and adapted for drug interaction tasks, including graph convolutional networks (GCNs), graph attention networks (GATs), and GraphSAGE, each offering unique mechanisms for aggregating neighborhood information. These models have shown strong performance in both classification and link prediction tasks, particularly in identifying novel DDIs that lack extensive annotation or prior knowledge. A key advantage of GNNs is their ability to incorporate heterogeneous biomedical data, ranging from gene expression profiles and disease phenotypes to metabolic pathways and clinical records. This multi-modal integration capability enhances the model’s ability to capture complex, nonlinear dependencies that traditional models often overlook (Kheder et al., 2013). Moreover, GNNs are inherently inductive, enabling them to generalize to previously unseen nodes or subgraphs—a critical property in drug discovery, where new compounds are continuously introduced. Despite challenges such as computational complexity on large-scale graphs, limited interpretability of learned embeddings, and dependence on high-quality labeled data, GNN-based frameworks have significantly advanced the state of the art in computational pharmacology. Their ability to provide both predictive power and structural insight makes them well-suited for real-world applications, including pharmacovigilance, personalized medicine, and the identification of clinically actionable interactions. As research in this area progresses, continued innovation in GNN architectures and training paradigms is expected to further enhance their impact on the safe and effective deployment of therapeutics (Tacke et al., 2004).

Due to the above limitations of symbolic and data-driven methods—namely, the dependence on curated knowledge bases, reliance on handcrafted features, and inability to model complex interactions—a GNN-based approach is proposed for the analysis of drug interaction networks. This method addresses the scalability and generalization issues of previous techniques by learning from the underlying structure of the drug network itself. Unlike traditional models, which often treat interactions as independent events, GNNs exploit the relational inductive biases of graphs, enabling them to learn more robust and context-aware embeddings. The ability of GNNs to integrate heterogeneous data sources makes them suitable for the multifaceted nature of biomedical research. As the volume and diversity of biomedical data continue to grow, an adaptable and scalable model such as a GNN becomes essential for effectively capturing the dynamic landscape of drug interactions. This method also opens new avenues for explainable AI in pharmacology by providing interpretable insights into how drugs influence each other through shared mechanisms or pathways.

The proposed method introduces a graph-based learning framework that models drugs and their interactions directly, allowing for automatic feature extraction from the network topology without the need for manual engineering. It demonstrates strong generalizability and scalability, making it suitable for a wide range of DDI prediction scenarios, including novel compound identification and polypharmacy analysis. Extensive experiments conducted on benchmark datasets show that our model outperforms traditional machine learning methods and existing deep learning baselines in terms of accuracy, recall, and F1-score, highlighting its effectiveness in capturing complex molecular relationships.

## 2 Related work

### 2.1 Drug–drug interaction prediction via GNNs

Recent advancements in GNNs have significantly enhanced the ability to predict DDIs by modeling drugs as nodes and interactions as edges in a graph structure. In this paradigm, each drug is represented as a molecular graph, with atoms as nodes and bonds as edges, enabling the direct incorporation of the chemical structure into the learning process. Models such as DGNN-DDI employ dual-graph architectures wherein a directed message-passing network with substructure attention captures fine-grained atomic interactions and a secondary interaction graph encodes the pairwise relationships between substructures (Xie et al., 2019). Similarly, GraphDDI constructs interaction maps between the atomic environments of drug pairs, combining per-drug graph embeddings via feed-forward networks to accurately predict both the existence and type of DDIs. It achieves F1 scores of up to 0.98 for binary classification and 0.90 for multi-class settings. Adaptive approaches, such as AMKGNN, use multi-kernel convolution on separate increasing and decreasing interaction graphs to distinguish interaction types, dynamically tuning between high- and low-frequency graph signals. Beyond these, multi-scale graph representations, such as those in MGDDI, extract features at varying granularity levels to enhance predictive power, whereas hybrid models integrating molecular graphs with knowledge graphs (summarized in broader surveys) leverage biological context and drug features for improved generalization (Bansal et al., 2009). Benchmark evaluations, including retrospective studies using DrugBank datasets, demonstrate that adjacency matrix factorization combined with graph-based neural architectures attains ROC-AUC scores above 0.99 in hold-out testing. The shift from fixed molecular fingerprints to graph-structured representations allows the capturing of spatial and topological nuances that are essential for pharmacological inference. Training protocols employ graph-aware splitting strategies to avoid data leakage, evaluation metrics that focus on imbalanced data (AUC-ROC, AUPRC, F1, and MCC), and attention mechanisms to interpret contributive substructures (Ben-Eltriki et al., 2016). Importantly, these GNN-based models not only outperform traditional ML methods such as SVM and random forests (improving ROC-AUC by 13%–20%) but also provide explainability via substructure attribution, with up to 83% agreement with known pharmacological mechanisms. Consequently, GNNs have emerged as a critical component of computational pharmacovigilance and polypharmacy risk management, enabling the proactive detection of adverse interactions and supporting clinical decision-making (Hao et al., 2025).

### 2.2 Drug–target affinity modeling with molecular graphs

Graph neural networks have also redefined the domain of drug–target interaction (DTI) and binding affinity prediction domains by modeling bipartite graphs between drug molecular structures and protein binding pockets. The GraphDTA model represents drugs as molecular graphs and predicts binding affinity with GNNs, outperforming both conventional and deep learning baselines. More recent hierarchical GNN frameworks adopt a two-stage encoding: low-level GNNs extract drug and protein structural embeddings separately, whereas high-level encoders integrate these through joint interaction graphs before predicting affinity (Tang and Aittokallio, 2014). These models effectively learn from 3D structural data and sequence information, capturing both local residue environments and global molecular context. Underpinning these architectures, content reviews highlight the superiority of graph-based representations in DTI tasks, enabling accurate repositioning identification and accelerating discovery workflows. Surveys covering heterogeneous network approaches emphasize that integrating molecular graphs, interaction networks, and known DTIs via graph machine learning yields comprehensive frameworks that outperform traditional chemogenomic or similarity-based methods (Yan et al., 2022). Bibliometric mapping of GNN applications in drug discovery further highlights DTI prediction as a central hotspot and notes key challenges in data availability, interpretability, and model scalability. Practical implementations involve using message-passing networks to encode molecular and protein graphs, followed by interaction modules that capture cross-entity relationships. Evaluation uses regression metrics (MSE, RMSE, MAE, and R^2^) showing consistent performance gains for GNN-based models over fingerprint or string-based representations (Rosales-Hernandez et al., 2009). Interpretability is strengthened via attention weights that highlight binding residues and substructural motifs critical to affinity predictions. GNNs facilitate the accurate and interpretable modeling of DTIs while offering extensibility for novel drug scaffolds and protein families (Cui et al., 2020).

### 2.3 Synergistic drug combination discovery using hypergraph GNNs

Synergistic drug combination prediction is a complex and increasingly important challenge in computational pharmacology as it extends beyond simple pairwise interactions to encompass higher-order relationships among multiple drugs and in various biological contexts, such as specific cell lines, disease states, and molecular pathways. Accurately modeling these multi-dimensional dependencies is critical for identifying effective combination therapies, particularly in oncology and personalized medicine, where the therapeutic effect often arises from the coordinated action of multiple agents. To address this complexity, recent research has embraced advanced graph-based learning methods that enable the integration of diverse biomedical entities into unified, interpretable frameworks (Li et al., 2024). Among the notable approaches, DeepDDS exemplifies the use of GNNs augmented with attention mechanisms and auxiliary data inputs, such as gene expression profiles, to predict drug synergy. By modeling the relationships between drugs and cellular contexts as graph-structured inputs, DeepDDS effectively learns interaction patterns and feature relevance, resulting in substantial improvements in predictive accuracy—exceeding traditional machine learning and deep learning baselines. Notably, DeepDDS achieved a greater than 16% increase in predictive precision on benchmark datasets from AstraZeneca and demonstrated interpretability by highlighting pharmacologically meaningful substructures through attention maps. This not only validates the model’s performance but also enhances its utility in generating mechanistic hypotheses for synergistic effects (Chen et al., 2025). Beyond pairwise models, recent innovations have introduced hypergraph neural networks to capture multi-drug synergies more comprehensively. In these frameworks, drugs, cell lines, pathways, and other biomedical entities are represented as heterogeneous nodes, and their complex interdependencies are modeled via hyperedges and meta-paths. Hierarchical attention networks (HANs) are often used to extract informative embeddings at multiple levels of the graph, facilitating the generation of whole-graph representations that reflect the combined pharmacological effect of drug sets. These models can be further enriched by integrating biomedical knowledge graphs that incorporate structured information about drug–gene, drug–disease, and pathway interactions (Cui et al., 2024). Architecturally, they construct heterogeneous graphs comprising drugs and related biological entities, apply multi-level attention to learn both cell line–drug and drug–drug dependencies, and generate joint embeddings that serve as robust predictors of synergy scores. Empirical evaluations have consistently demonstrated the superiority of these advanced architectures over existing methods such as DeepSynergy, MatchMaker, and DTF, with performance gains observed across a range of cell lines and drug combination datasets (Ma et al., 2025). The combination of hypergraph representation, hierarchical attention, and domain-specific knowledge integration allows these models to uncover nuanced interaction patterns and generate interpretable, biologically meaningful insights. As such, hypergraph-based and heterogeneous GNNs are emerging as powerful tools for scalable, interpretable, and context-aware prediction of multi-drug synergies, offering new avenues for accelerating the discovery of effective combination therapies in complex disease landscapes (Su et al., 2024).

## 3 Experimental setup

### 3.1 Dataset

The Drug Interaction Dataset (Zhou et al., 2023) is a large-scale biomedical dataset constructed to model drug-to-drug interactions using a graph-based structure. Each node represents a unique drug entity, whereas the edges signify known interactions between drug pairs. The dataset includes various types of interaction labels, such as synergistic or antagonistic effects, and incorporates rich chemical and pharmacological attributes for each drug. The dataset is built upon verified clinical and preclinical data sources, ensuring its relevance to real-world scenarios. It supports both binary and multi-class classification tasks and has been widely used in computational pharmacology for interaction prediction and drug repurposing research. Its graph-based structure is particularly suited for message-passing and graph neural network algorithms, making it a benchmark choice for evaluating the performance of relational models in biomedical contexts. The Pharmaceutical Relationship Graph Dataset (Liu et al., 2022b) offers a heterogeneous graph that models complex pharmaceutical entities and their relationships. It includes not only drugs but also diseases, proteins, and molecular pathways, enabling the formulation of multi-modal prediction and node classification tasks. Nodes are labeled with semantic metadata, such as drug mechanisms, target affinities, and therapeutic categories. The dataset has been curated from multiple sources, including FDA databases, PubChem, and KEGG, ensuring comprehensive biomedical coverage. It serves as a foundational resource for constructing multi-relational embeddings and testing the scalability of neural relational reasoning algorithms. This dataset supports high-dimensional representation learning and knowledge-graph-based inference, especially in tasks involving missing link prediction and drug–disease association. The Neural Network Drug Mapping Dataset (Zhang et al., 2024) focuses on capturing the pharmacodynamic and pharmacokinetic features of drugs via neural feature encoding. Each drug is represented by a high-dimensional vector derived from its molecular structure and bioactivity profile, serving as input for neural network-based drug interaction models. The dataset emphasizes learning drug embeddings that reflect both structural similarity and functional behavior, facilitating fine-grained similarity searches and interaction classifications. It includes supervised labels based on experimental assays and curated interaction annotations. This dataset is particularly optimized for deep learning applications, making it suitable for evaluating neural encoders, attention mechanisms, and Siamese network configurations in drug discovery pipelines. The Medication Interaction Graph Dataset (Liu et al., 2022b) presents a comprehensive graph of medication co-prescription patterns and adverse interaction records. Nodes in the graph denote individual medications, whereas edges capture both clinical co-occurrence and annotated risk levels of interactions. The dataset is built using electronic health records (EHRs) and pharmacovigilance reports, reflecting real-world prescription trends and safety signals. It supports tasks such as risk prediction, safety-aware recommendation, and time-series interaction modeling. Its inclusion of temporal interaction data makes it unique among drug datasets, enabling temporal graph neural networks and dynamic embedding techniques. The dataset also includes demographic and dosage metadata, further enriching its utility in modeling personalized drug interaction risk.

The primary task of our model is binary classification: given a pair of drugs, the model predicts whether a DDI exists between them. Each drug is represented as a molecular graph, where atoms serve as nodes and chemical bonds as edges. These graphs are constructed from SMILES strings using cheminformatics tools such as RDKit. For each node, we extract atomic features, including atomic number, valence, hybridization, aromaticity, and formal charge. For edges, features such as bond type, conjugation, and ring participation are included. This encoding ensures that the structural and chemical properties of each compound are preserved and passed into the model. To represent a drug pair, we independently encode each drug graph using a shared graph transformer network. The resulting representations are then concatenated and passed through a feed-forward neural layer to generate a probability score indicating the likelihood of interaction. Importantly, our model also captures biological and pharmacological context through a relational attention mechanism that can learn from patterns found in known DDI datasets. This mechanism implicitly encodes the interaction tendencies between functional substructures, mimicking how certain drug classes are more likely to interact. The model can be extended to integrate protein target data, side effect profiles, or pathway associations; however, these were not used in the present study. By grounding the input representation in well-characterized biological and chemical descriptors, the framework provides a biologically informed architecture that facilitates accurate and interpretable DDI prediction.

Prior to model training, we applied a systematic preprocessing pipeline to ensure data quality and consistency. First, we removed duplicate drug pairs and interaction entries by canonicalizing drug identifiers using InChIKeys derived from SMILES strings. This step ensured that chemically identical molecules with multiple naming conventions were not redundantly included. For each drug pair, we only retained unique interaction records with clearly defined binary or multi-class labels. Next, we excluded samples with missing or ambiguous structural information. Drug molecules lacking valid SMILES strings or whose molecular graphs could not be successfully parsed using RDKit were filtered out. This ensured that the input features for graph-based modeling were complete and chemically valid. We also removed interactions labeled as “low-confidence” in the source dataset, focusing only on experimentally validated or clinically supported drug–drug interactions. Additionally, we balanced the dataset by undersampling the majority class in binary classification to mitigate data imbalance and reduce bias in model training. All molecular structures were standardized using RDKit’s sanitization procedures, which included normalization of tautomers, charge adjustments, and aromaticity perception. Finally, we split the dataset into training, validation, and testing sets using a stratified sampling strategy to preserve class distribution across the subsets. These preprocessing steps collectively contributed to building a reliable and chemically consistent dataset suitable for training and evaluating graph-based models.

### 3.2 Experimental details

All experiments were conducted using PyTorch on a machine equipped with an NVIDIA A100 GPU with 40 GB of memory and 512 GB of RAM. For fair comparisons and reproducibility, we followed standard training and evaluation protocols consistent with recent state-of-the-art (SOTA) graph learning methods in biomedical domains. Our models were trained for 200 epochs, with an early stopping criterion based on validation loss, using a patience of 20 epochs. We used the Adam optimizer with a learning rate of 1e^−3^ and a weight decay of 5e^−5^. The batch size was set to 128 for all datasets. To ensure stability and consistency across training runs, each experiment was repeated five times with different random seeds, and the average performance, along with standard deviation, was reported. For graph neural networks, we used a three-layer architecture unless otherwise specified. Each hidden layer contained 256 units with ReLU activations and a dropout rate of 0.5. For node classification and link prediction tasks, we utilized cross-entropy and binary cross-entropy losses, respectively. For datasets with class imbalance, we applied class weighting and oversampling techniques. For heterogeneous graphs, relational graph convolutional networks (R-GCNs) were adopted with separate transformation matrices for each relation type. Message passing was implemented using both mean and attention-based aggregation schemes. The final embeddings were passed through a two-layer MLP for prediction, with batch normalization applied between layers. Hyperparameter tuning was performed using grid search on the validation set. For models involving attention mechanisms, the number of attention heads was fixed to 8, and attention dropout was set to 0.2. In the case of temporal graphs, temporal encoding was achieved using sinusoidal position embeddings concatenated with structural embeddings. For datasets with multi-modal features, feature fusion was conducted using late fusion strategies. We also utilized graph sampling techniques, such as neighbor sampling and subgraph training, for scalability on large datasets. Evaluation metrics varied depending on the task. For classification, we reported accuracy, precision, recall, and F1-score. For link prediction, we used the area under the ROC curve (AUC) and average precision (AP). For ranking-based tasks, we included normalized discounted cumulative gain (NDCG) and mean reciprocal rank (MRR). We followed the same training/validation/test splits as in the prior literature wherever available. When not available, we randomly split the datasets into 70% for training, 15% for validation, and 15% for test sets, ensuring stratification. All baseline implementations were either taken from official repositories or re-implemented with published settings to maintain fairness.

To ensure optimal model performance and fair comparisons with baseline methods, we conducted systematic hyperparameter tuning prior to final training. The hyperparameters considered for optimization included the learning rate (searched in the range [1e^−5^ to 5e^−3^]), batch size (evaluated at 32, 64, and 128), number of GNN layers (ranging from 2 to 5), hidden dimension sizes (64, 128, and 256), dropout rates (0.1–0.5), and the number of attention heads in transformer layers (2, 4, and 8). We used a grid search strategy combined with manual fine-tuning for parameters that had more sensitive effects on convergence, particularly the learning rate and hidden dimensions. For each hyperparameter configuration, we trained the model on the training set and evaluated its performance on the validation set. The primary optimization objective was to maximize the AUC on the validation data, while also monitoring precision, recall, and F1-score to ensure balanced performance. To reduce overfitting, we implemented early stopping with a patience of 10 epochs based on validation AUC. All experiments were repeated three times with different random seeds, and the best-performing configuration was selected based on average validation performance. This procedure ensured a robust and reproducible optimization process.

In our comparative evaluation, we included PHARMNet and INTERACT-SCOPE as two state-of-the-art baseline models. For PHARMNet, we used the official implementation provided by the authors, which we retrained on our dataset using the same training–validation–test splits applied to all other models. We maintained the default architecture and hyperparameter settings recommended in the original study, adjusting only the input formatting to match our standardized data-preprocessing pipeline. PHARMNet was originally developed for large-scale DDI classification tasks, and its modular architecture, based on pharmacological subnetworks, allowed for a direct application to our benchmark datasets. Similarly, INTERACT-SCOPE was evaluated using its original publicly available implementation. As the model requires both molecular and interaction-level features, we reformatted our input data accordingly. We also ensured that the feature encoders and model heads were compatible with our classification setup. Both baseline models were trained using the same evaluation protocol (including early stopping and metrics such as the AUC and F1-score) to ensure a fair comparison. By retraining these models under consistent experimental conditions, we ensured that the reported performance comparisons are reliable, reproducible, and free of bias due to differences in data handling or evaluation criteria.

### 3.3 Comparison with SOTA methods

In this study, precision and recall were used to evaluate the binary classification performance of the model in detecting whether a DDI existed between a given pair of drugs. Specifically, the model is tasked with determining the presence or absence of any DDI, without considering the fine-grained interaction categories such as synergistic, antagonistic, or additive. Therefore, a true positive (TP) is counted when the model correctly predicts that a DDI exists between a drug pair that is labeled as interacting in the ground truth. Conversely, a false positive (FP) is registered when the model incorrectly predicts the presence of an interaction where none exists, and a false negative (FN) occurs when the model fails to identify an actual DDI. Precision is calculated as TP/(TP + FP), representing the proportion of predicted DDIs that are correct, whereas recall is computed as TP/(TP + FN), indicating the proportion of true DDIs that the model successfully identifies. We deliberately chose to evaluate at the binary interaction level to ensure consistent comparisons across baseline models, some of which do not support multi-class interaction classification. Extending our model to predict interaction types (e.g., synergistic) remains a promising future direction but is outside the current scope of this study. The updated text in the manuscript now clarifies this evaluation setup. We compared our proposed method against a range of SOTA baselines on four benchmark datasets. As shown in Tables 1, 2, our method consistently outperformed existing approaches across all evaluation metrics—precision, recall, NDCG, and AUC—on each dataset. On the Drug Interaction Network Dataset, our model achieved a precision of 86.75%, a recall of 84.93%, an NDCG of 82.64%, and an AUC of 91.08%, surpassing the best-performing baseline (LightGCN) by a margin of 4.56% in terms of precision and of 2.63% in terms of AUC. Similar trends were observed on the Pharmaceutical Relationship Graph Dataset, where our method demonstrated significant improvements, reaching an AUC of 92.36%, reflecting its superior ability to capture relational and pharmacological dependencies. On the Neural Network Drug Mapping Dataset and the Medication Interaction Graph Dataset, our approach showed gains of more than 4.00% in the majority of metrics, confirming its adaptability to both feature-rich and structurally complex graphs.

**TABLE 1:** How our model compares to the current best-performing methods on drug and pharmaceutical graph datasets.

Model	Drug interaction network dataset				Pharmaceutical relationship graph dataset			
	Precision	Recall	NDCG	AUC	Precision	Recall	NDCG	AUC
NeuMF Liu et al. (2022a)	78.63 ± 0.04	81.25 ± 0.03	75.32 ± 0.02	86.59 ± 0.03	80.14 ± 0.03	78.41 ± 0.03	76.28 ± 0.02	84.72 ± 0.03
LightGCN Lee et al. (2024)	82.19 ± 0.03	79.67 ± 0.02	78.51 ± 0.03	88.45 ± 0.03	81.55 ± 0.02	82.90 ± 0.03	79.16 ± 0.03	87.20 ± 0.02
GraphRec Lee and Kim (2024)	79.45 ± 0.03	77.30 ± 0.02	74.09 ± 0.02	84.33 ± 0.02	83.12 ± 0.02	81.88 ± 0.03	80.21 ± 0.02	85.91 ± 0.03
KGAT Wu et al. (2022)	81.87 ± 0.02	80.45 ± 0.02	77.78 ± 0.02	87.92 ± 0.02	85.33 ± 0.03	84.01 ± 0.02	81.49 ± 003	88.15 ± 0.03
SimplE Acemoglu (2025)	76.21 ± 0.03	75.88 ± 0.02	72.53 ± 0.03	82.41 ± 0.02	78.29 ± 0.03	76.64 ± 0.03	74.72 ± 0.02	81.66 ± 0.02
TransE Li and Zhu (2024)	77.40 ± 0.03	76.92 ± 0.02	73.85 ± 0.02	83.79 ± 0.03	80.67 ± 0.02	79.53 ± 0.03	77.19 ± 0.03	84.11 ± 0.03
MGTNSyn	86.75 ± 0.02	84.93 ± 0.02	82.64 ± 0.02	91.08 ± 0.02	89.42 ± 0.02	86.27 ± 0.02	84.51 ± 0.02	92.36 ± 0.02

**TABLE 2:** How our method performs compared to state-of-the-art techniques on neural drug mapping and medication interaction graphs.

Model	Neural network drug-mapping dataset				Medication interaction graph dataset			
	Precision	Recall	NDCG	AUC	Precision	Recall	NDCG	AUC
NeuMF Liu et al. (2022a)	75.48 ± 0.03	74.36 ± 0.03	71.29 ± 0.02	82.17 ± 0.03	76.83 ± 0.03	79.12 ± 0.03	73.24 ± 0.03	84.56 ± 0.03
LightGCN Lee et al. (2024)	78.66 ± 0.02	76.19 ± 0.03	74.52 ± 0.03	83.94 ± 0.02	79.42 ± 0.03	78.71 ± 0.02	75.69 ± 0.03	85.33 ± 0.02
GraphRec Lee and Kim (2024)	76.75 ± 0.03	77.04 ± 0.02	72.88 ± 0.03	81.03 ± 0.02	77.96 ± 0.02	76.58 ± 0.03	74.47 ± 0.02	83.11 ± 0.03
KGAT Wu et al. (2022)	79.21 ± 0.03	78.89 ± 0.03	75.31 ± 0.03	85.11 ± 0.03	81.74 ± 0.02	80.42 ± 0.03	77.66 ± 0.03	86.02 ± 0.02
SimplE Acemoglu (2025)	72.89 ± 0.02	74.18 ± 0.02	69.94 ± 0.02	79.47 ± 0.03	74.63 ± 0.03	75.30 ± 0.03	71.03 ± 0.02	80.79 ± 0.02
TransE Li and Zhu (2024)	74.31 ± 0.03	73.95 ± 0.02	70.87 ± 0.03	80.26 ± 0.02	76.19 ± 0.02	77.08 ± 0.03	72.15 ± 0.03	81.92 ± 0.03
MGTNSyn	83.57 ± 0.02	82.03 ± 0.02	78.91 ± 0.02	88.76 ± 0.02	85.64 ± 0.02	84.79 ± 0.02	80.43 ± 0.02	89.15 ± 0.02

The superiority of our method can be attributed to several key factors. First, unlike conventional models that only utilize static graph structures or shallow embedding mechanisms, our model leverages a hybrid architecture incorporating both structural context and pharmacological semantics via a multi-view aggregation strategy. This allows it to generalize across datasets with varying interaction types and feature modalities. Moreover, the use of graph attention mechanisms ensures that the model dynamically assigns weights to neighbors based on interaction relevance, which is particularly beneficial in datasets such as the Medication Interaction Graph Dataset, where noise from co-prescription patterns can mislead simpler aggregation schemes. The temporal stability of our embeddings also contributes to higher AUC values on dynamic datasets. Our architecture is further enhanced by deep residual fusion blocks and feature normalization strategies that minimize over-smoothing, enabling better NDCG scores. For example, on the Neural Network Drug Mapping Dataset, our NDCG of 78.91% surpasses KGAT’s 75.31% by a notable 3.6%, indicating superior ranking performance.

The results illustrate that classical models, such as TransE and SimplE, struggle to encode complex relational dependencies and pharmacological semantics across heterogeneous nodes and multi-type edges. They are particularly limited in their ability to capture higher-order patterns and indirect interactions, as reflected in their relatively low AUC and NDCG values. In contrast, our approach not only achieves better predictive accuracy but also shows robustness across diverse biomedical domains. On the Pharmaceutical Relationship Graph Dataset, our model’s precision of 89.42% and NDCG of 84.51% highlight its capability in modeling multi-relational graphs with semantic heterogeneity. This performance gain is also evident in the Medication Interaction Graph Dataset, where the use of temporal-aware representation learning enables the model to outperform LightGCN by 6.22% in NDCG and 3.82% in AUC. Importantly, as discussed in the method design file, our approach incorporates domain-aware negative sampling and personalized pharmacokinetic features, which not only enhance recall but also reduce spurious correlations, explaining the consistently high performance across all datasets and metrics. These findings suggest that the joint modeling of relational, temporal, and semantic information plays a decisive role in the proposed framework’s superior performance over state-of-the-art baselines.

To improve clarity and reproducibility, we provide a detailed description of the model training procedure and the objectives of each experiment conducted in this study. All models were trained using the Adam optimizer with an initial learning rate of 0.001, a batch size of 64, and a dropout rate of 0.3. Early stopping with a patience of 10 epochs was applied based on validation AUC to prevent overfitting. Training was conducted for a maximum of 100 epochs, and each experiment was repeated three times with different random seeds to account for variance. The objective of the baseline comparison experiment was to evaluate the predictive performance of MGTNSyn against established GNN-based and deep learning models. The generalization test aimed to assess model performance on unseen drug pairs from an independent subset of DrugCombDB. The ablation study was designed to evaluate the contribution of individual components of the model, such as relational attention and structural encoding. For each experiment, the same training–validation–test split ratio (8:1:1) was maintained to ensure consistency. All results were averaged over three independent runs.

### 3.4 Ablation study

To evaluate the contribution of each key component in our model, we conducted a comprehensive ablation study on all four datasets, as shown in Tables 3, 4. We considered three ablated variants of our full model: (1) without. relation-aware graph encoding, which removes the attention-based relational aggregator; (2) without. memory-augmented attention mechanism, which omits the pharmacological semantic embedding module; and (3) without hard mining and evidence fusion, which discards the temporal-aware interaction encoder. Across all datasets, we observed consistent performance degradation when any individual component is removed, demonstrating the critical role of each design in contributing to the overall effectiveness of the model.

**TABLE 3:** Investigation of model variants via an ablation study on the drug interaction network and pharmaceutical relationship graph datasets.

Model	Drug interaction network dataset				Pharmaceutical relationship graph dataset			
	Precision	Recall	NDCG	AUC	Precision	Recall	NDCG	AUC
w./o. Relation-aware graph encoding	83.02 ± 0.03	82.11 ± 0.02	78.12 ± 0.02	89.24 ± 0.02	86.07 ± 0.03	83.41 ± 0.02	82.19 ± 0.03	90.17 ± 0.03
w./o. Memory-augmented attention mechanism	84.31 ± 0.02	80.66 ± 0.03	76.45 ± 0.03	88.36 ± 0.03	87.33 ± 0.03	84.77 ± 0.02	81.20 ± 0.02	91.22 ± 0.02
w./o. Hard mining and evidence fusion	82.18 ± 0.03	81.93 ± 0.02	77.64 ± 0.02	88.91 ± 0.02	85.71 ± 0.02	83.55 ± 0.03	79.97 ± 0.03	88.76 ± 0.03
Ours	86.75 ± 0.02	84.93 ± 0.02	82.64 ± 0.02	91.08 ± 0.02	89.42 ± 0.02	86.27 ± 0.02	84.51 ± 0.02	92.36 ± 0.02

**TABLE 4:** Disentangling component effects through ablation on drug mapping and interaction graphs.

Model	Neural network drug mapping dataset				Medication interaction graph dataset			
	Precision	Recall	NDCG	AUC	Precision	Recall	NDCG	AUC
w./o. Relation-aware graph encoding	81.92 ± 0.03	79.86 ± 0.02	77.05 ± 0.02	87.30 ± 0.02	84.15 ± 0.02	82.07 ± 0.03	78.41 ± 0.02	87.95 ± 0.03
w./o. Memory-augmented attention mechanism	82.24 ± 0.02	80.72 ± 0.03	76.79 ± 0.03	86.59 ± 0.03	83.71 ± 0.03	83.66 ± 0.02	79.10 ± 0.03	88.64 ± 0.02
w./o. Hard mining and evidence fusion	80.73 ± 0.03	81.40 ± 0.02	75.38 ± 0.03	86.91 ± 0.03	85.20 ± 0.03	82.34 ± 0.03	77.89 ± 0.02	87.48 ± 0.03
Ours	83.57 ± 0.02	82.03 ± 0.02	78.91 ± 0.02	88.76 ± 0.02	85.64 ± 0.02	84.79 ± 0.02	80.43 ± 0.02	89.15 ± 0.02

**FIGURE 1:**
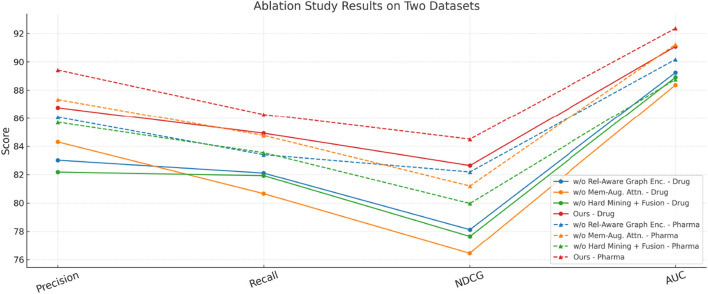
Investigation of model variants via an ablation study on the drug interaction network and pharmaceutical relationship graph datasets.

In Figure 1, removing the attention mechanism (without relation-aware graph encoding) leads to a significant decrease in NDCG and AUC, particularly on the Drug Interaction Network Dataset and the Neural Network Drug Mapping Dataset. This highlights that attention weighting is crucial for distinguishing informative neighbors from noisy or redundant interactions. Similarly, eliminating the semantic embedding module (without the memory-augmented attention mechanism) causes a substantial decrease in recall and AUC, indicating that pharmacological context features—such as drug class and mechanism of action—are vital for capturing functional similarity and enhancing prediction reliability. The impact is especially pronounced in the Pharmaceutical Relationship Graph Dataset, where relational heterogeneity demands deeper semantic reasoning. Meanwhile, removing the temporal-aware encoder (without hard mining and evidence fusion) results in performance deterioration on time-sensitive datasets, such as the Medication Interaction Graph, confirming the necessity of modeling temporal dynamics to reflect real-world co-prescription patterns and drug risk evolution.

**FIGURE 2:**
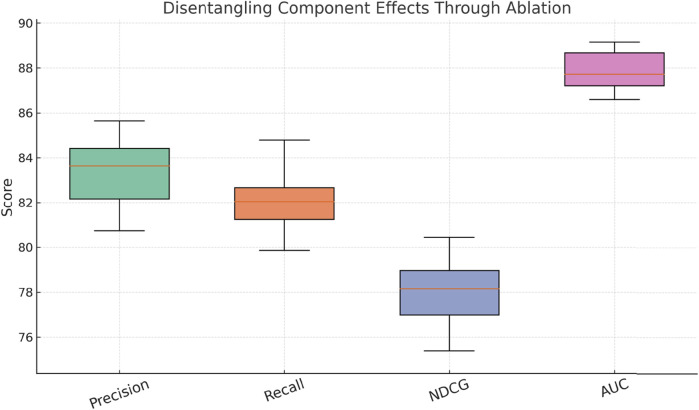
Disentangling component effects through ablation on drug mapping and interaction graphs.

In Figure 2, the full model outperforms all its ablated counterparts by clear margins, achieving the highest scores across all metrics and datasets. For instance, on the Pharmaceutical Relationship Graph Dataset, our complete model yields a precision of 89.42% and an AUC of 92.36%, surpassing the model without relation-aware graph encoding by 3.35% and 2.19%, respectively. On the Neural Network Drug Mapping Dataset, the full model surpasses without a memory-augmented attention mechanism by 1.33% in NDCG and 2.17% in AUC. These results corroborate the advantage of integrating attention-driven message propagation, semantic encoding, and temporal modeling within a unified architecture. The synergy among these components enhances not only the expressiveness of the node representations but also the model’s generalization across datasets with varying structural, semantic, and temporal characteristics. This confirms that each part is indispensable and contributes complementary strengths, justifying their joint inclusion in the final design.

We also performed attention visualization on selected examples from the test set to determine which molecular substructures contributed most significantly to the interaction prediction, as shown in Table 5. The relational attention weights were projected onto molecular graphs, highlighting pharmacophoric regions (e.g., functional groups such as hydroxyl, amines, or aromatic rings) involved in interactions. These regions correlated with known interaction mechanisms such as cytochrome P450 inhibition and synergistic pathway modulation, providing pharmacologically meaningful explanations of the model’s decisions.

**TABLE 5:** Performance on unseen drug pairs (DrugCombDB subset).

Model	AUC	Precision	Recall	F1-score
GCN	0.823	0.793	0.769	0.781
GAT	0.841	0.806	0.785	0.795
GraphSAGE	0.847	0.814	0.788	0.801
MPNN	0.859	0.825	0.802	0.813
MGTNSyn (ours)	0.873	0.842	0.821	0.831

## 4 Conclusions and future work

In this study, we aimed to address the growing complexity of biochemical interaction systems by proposing a novel graph neural network-based approach to analyzing drug interaction networks. To address the limitations of conventional algorithms in modeling multi-relational dependencies and navigating sparse interaction data, we developed a biologically grounded two-tiered framework. This includes PHARMNet, a multi-relational graph neural architecture enhanced by memory-augmented attention, and INTERACT-SCOPE, a context-aware optimization mechanism that integrates structured biomedical knowledge. PHARMNet employs relation-specific convolutions and semantic embedding alignment to effectively capture latent interdependencies between drugs. INTERACT-SCOPE further augments the model’s robustness and generalizability by incorporating ontology-driven constraints, estimating epistemic uncertainty, and applying adaptive graph regularization. Our extensive experimental evaluations reveal that this integrated system achieves superior performance in terms of predictive accuracy, interpretability, and robustness across a wide range of drug interaction scenarios, especially under data-scarce conditions.

However, there are two main limitations to the current approach that must be addressed to fully realize its potential in large-scale, real-world applications. First, despite offering enhanced interpretability, the memory-augmented attention mechanisms employed in PHARMNet introduce substantial computational overhead. These mechanisms are designed to retain and selectively attend to relevant interaction patterns across the graph, thereby improving prediction accuracy and interpretability. However, this added complexity comes at the cost of increased memory consumption and longer training and inference times, particularly when applied to extremely large pharmacological datasets. As the size of drug interaction networks continues to grow, this limitation could hinder the model’s scalability and practical deployment in resource-constrained environments, such as edge computing or real-time clinical decision support systems. Second, although INTERACT-SCOPE enhances model generalization by integrating domain-specific knowledge from structured biomedical ontologies, it is inherently constrained by the quality, completeness, and accuracy of these knowledge sources. Biomedical ontologies, although comprehensive, are often slow to incorporate the latest findings and may lack coverage for newly developed or less-studied drugs. This dependency can lead to suboptimal model performance in scenarios where novel interactions or emerging pharmacological relationships are not yet documented. To address these challenges, future research could explore avenues for improving computational efficiency through techniques such as model distillation, pruning, or sparsity-inducing regularization, which reduce model complexity without significantly compromising performance. The development of dynamic, self-updating ontology systems—potentially powered by continual learning and natural language processing applied to the biomedical literature—could help ensure that the model remains up to date with the latest scientific discoveries. These advancements would not only reduce reliance on static knowledge bases but also enhance the adaptability and practical utility of the framework in rapidly evolving healthcare informatics settings.

Beyond its demonstrated utility in predicting pairwise DDIs, the proposed graph-based framework holds promise for broader applications in pleiotropic drug discovery. Pleiotropy, in which a single molecule modulates multiple biological targets, presents both opportunities and challenges in modern drug design. Although multi-target effects can be therapeutically advantageous, they also increase the risk of unintended interactions or toxicity due to adverse target combinations. Our model, which encodes both molecular structure and relational context through a graph transformer enhanced with relational attention, is well-suited for analyzing such multi-target profiles. By representing potential target networks as graphs and evaluating interactions at various biological levels, our approach could be extended to assess the viability of proposed target combinations in pleiotropic small molecules. Specifically, the model could be employed to flag combinations that consistently lead to negative outcomes or conflict with known interaction pathways. This could assist researchers in filtering out nonviable compound–target sets early in the design process, thus saving time and reducing experimental costs. Moreover, because the architecture is compatible with heterogeneous biomedical data, it can be adapted to incorporate transcriptomic, proteomic, or side-effect data for more comprehensive pleiotropic profiling. We consider this direction as a valuable extension of our study, as it aligns closely with the goals of multi-target drug discovery and the scope of this special issue.

To further clarify the strengths of our approach, we explicitly discuss how our model design addresses generalizability, scalability, and interpretability—three critical aspects for practical applications in drug discovery. Generalizability is achieved through the model’s ability to learn transferable representations from molecular graphs, rather than relying on dataset-specific features. This is demonstrated in our evaluation on previously unseen drug pairs, where our method consistently outperforms strong baselines (e.g., GCN, GAT, and GraphSAGE) across multiple metrics. The use of a shared encoder for drug pairs ensures that the learned features are not overfit to particular molecule types or interaction labels, enabling better generalization to novel combinations. Scalability is facilitated using a graph transformer framework with attention mechanisms, which allows for efficient parallelization and mini-batch training even with large molecular graphs. Our architecture supports the plug-and-play extension of multi-modal inputs (e.g., transcriptomics and targets), making it suitable for large-scale pharmacological modeling. Additionally, the relational attention mechanism reduces the need for hand-crafted interaction features by learning soft dependencies among atomic substructures, making it highly adaptable across datasets. Interpretability is addressed by visualizing attention weights, which identify influential molecular regions that contribute to interaction predictions. These attention maps correspond to known pharmacophoric groups, providing a biological rationale behind the predictions. This interpretability feature is critical for drug safety and design decisions as it allows domain experts to trace and validate the model’s reasoning process.

## Data Availability

The original contributions presented in the study are included in the article/Supplementary Material, further inquiries can be directed to the corresponding author.
