# Supplementary material for: Analysis of the interaction network relationship between drugs using a graph neural network
Source: Front Pharmacol. 2026 Jun 12;17:1686243. doi: 10.3389/fphar.2026.1686243 (PMC13299097; doi:10.3389/fphar.2026.1686243)
Supplement: Supplementary file 1 [file Supplementaryfile1.pdf]

Analysis of the interaction network relationship between drugs using graph neural network

Overview

To bridge the gap between abstract graph representations and biologically meaningful modeling, our approach incorporates domain-specific chemical and biological information at multiple stages of the pipeline. First, each drug molecule is represented as a molecular graph where nodes correspond to atoms and edges to chemical bonds. These molecular graphs are constructed from SMILES (Simplified Molecular Input Line Entry System) strings using RDKit, which allows the precise extraction of atom-level and bond-level descriptors. For each atom, we encode features such as atomic number, degree, hybridization, formal charge, and aromaticity—attributes that directly influence molecular behavior and pharmacokinetics. Bond features include bond type (single, double, aromatic), conjugation, and ring membership, which are essential for understanding molecular stability and reactivity. Furthermore, biological interaction information is implicitly integrated via the edge-level attention mechanism, which learns to prioritize certain atom-atom interactions based on their contextual importance in known drug-drug interaction patterns. In cases where protein targets are available, the model architecture can be extended to jointly represent drug-target networks, enabling the inclusion of target pathway information and facilitating multi-modal learning. By embedding these domain-informed features into the graph transformer layers, the model gains the ability to reason over biologically plausible structures and mechanisms, beyond purely topological patterns. This strategy ensures that the model is not only mathematically expressive but also biologically grounded, which is critical for tasks such as predicting drug synergy or toxicity. The revised Method section now explicitly describes how this integration is achieved in practice.

In this section, we introduce the methodological framework for analyzing interaction network relationships between pharmacological compounds. Drug interaction networks have become a critical analytical structure in biomedical informatics, serving as a scaffold for understanding the complex relational patterns that emerge when multiple compounds exert influence across overlapping biological pathways. Our approach is grounded in the observation that DDIs are neither isolated nor merely pairwise phenomena; rather, they are embedded in a heterogeneous relational space influenced by molecular structure, therapeutic function, target affinity, and biological context.

Section formulates the problem by treating drugs as nodes in a heterogeneous interaction graph and defines the semantic and topological elements that govern this network, such as pharmacophore similarities, target co-affinity, and side-effect co-expression. To enable downstream learning and inference tasks, we formalize this interaction network as a multi-relational hypergraph $\mathcal{G} = (\mathcal{V}, \mathcal{E}, \mathcal{R})$, where $\mathcal{V}$ denotes the drug entities, $\mathcal{E}$ the edge types encoding interaction contexts, and $\mathcal{R}$ the set of relational types, including synergistic, antagonistic, and unknown interactions. Section introduces a novel representation learning architecture named PHARMNet (Pharmacological Relational Memory Network), which leverages both structural and semantic embeddings to predict latent interaction patterns. This model departs from conventional graph neural network paradigms by integrating a multi-perspective attention mechanism that jointly models drug similarity, target overlap, and indication context. The architecture is designed to capture higher-order dependencies within pharmacological subspaces, supporting both inductive prediction on unseen drug pairs and transductive reasoning over known entities. Section presents our strategic framework, INTERACT-SCOPE, a domain-informed strategy that guides representation refinement through

biochemical priors. This strategy addresses a key limitation in existing models—namely, the oversimplified assumption of uniformly distributed interactions—by introducing adaptive graph re-weighting based on curated ontologies such as DrugBank, SIDER, and MeSH. By integrating this curated knowledge with structural inference, INTERACT-SCOPE enhances the interpretability and reliability of predicted interactions, especially in underrepresented pharmacological categories. Collectively, these components establish a unified methodology for learning and reasoning over drug interaction networks, where both the topology and semantics of pharmacological data are jointly optimized. The overarching goal is to enable precise, scalable, and biologically informed inference of drug interaction effects, ultimately aiding in clinical decision support and drug repositioning. The subsequent sections expand on these components in detail, beginning with a formal definition of the problem and foundational notation.

Preliminaries

In this section, we present the formalization of the drug interaction network problem through a multi-relational and topologically-aware graph framework. Our objective is to capture both direct and indirect pharmacological interactions, enabling scalable reasoning across high-dimensional drug data. We begin by introducing the fundamental notations and mathematical structures used throughout the paper.

Let $\mathcal{D} = \{d_1, d_2, \ldots, d_N\}$ denote a set of N distinct drug compounds. Each drug d_i is associated with a vector of molecular features $\mathbf{x}_i \in \mathbb{R}^F$, where F is the number of structural or physicochemical descriptors. The pairwise interaction between two drugs (d_i, d_j) is not limited to binary presence or absence but is categorized into multiple relation types $\mathcal{R} = \{r_1, r_2, \ldots, r_K\}$ such as synergistic, antagonistic, neutral, or unknown.

We define the multi-relational drug interaction graph as,

$$\mathcal{G} = (\mathcal{V}, \mathcal{E}, \mathcal{R}), \tag{S1}$$

where $\mathcal{V} = \mathcal{D}$ represents the node set of drugs, and $\mathcal{E} \subseteq \mathcal{V} \times \mathcal{V} \times \mathcal{R}$ is the set of labeled edges representing known interactions.

Let $\mathbf{A}^{(r_k)} \in \{0,1\}^{N \times N}$ denote the adjacency matrix for relation type $r_k \in \mathcal{R}$. Then, the full interaction tensor can be denoted as,

$$\mathcal{A} \in \{0,1\}^{N \times N \times K}, \quad \mathcal{A}_{ijk} = 1 \iff (d_i, d_j, r_k) \in \mathcal{E}. \tag{S2}$$

The pharmacological similarity between drugs is modeled via a kernel function $S : \mathcal{D} \times \mathcal{D} \to [0,1]$, defined as,

$$S(d_i, d_j) = \exp\left(-\frac{\|\mathbf{x}_i - \mathbf{x}_j\|_2^2}{\sigma^2}\right), \tag{S3}$$

where σ is a bandwidth hyperparameter.

Each drug d_i is also associated with a set of therapeutic targets $\mathcal{T}_i \subseteq \mathcal{T}$, where $\mathcal{T}$ is the universe of protein targets. Define a binary target affinity matrix $\mathbf{Y} \in \{0,1\}^{N \times M}$, with $M = |\mathcal{T}|$, where,

$$\mathbf{Y}_{im} = \begin{cases} 1 & \text{if } t_m \in \mathcal{T}_i, \\ 0 & \text{otherwise.} \end{cases} \tag{S4}$$

We now define a joint semantic interaction score between drugs d_i and d_j,

$$\mathcal{J}(d_i, d_j) = \lambda_1 S(d_i, d_j) + \lambda_2 \cdot \text{Jaccard}(\mathcal{T}_i, \mathcal{T}_j), \tag{S5}$$

where $\lambda_1, \lambda_2 \geq 0$ are balancing coefficients.

Relational Pharmacological Embedding Network (PHARMNet)

In this section, we introduce PHARMNet (Pharmacological Relational Memory Network), a neural architecture designed to model the structural, relational, and semantic properties of drug interaction networks. PHARMNet captures higher-order relational dependencies through an integration of multi-relational graph encoding, memory-enhanced attention, and biochemical embedding regularization(As shown in Figure S1).

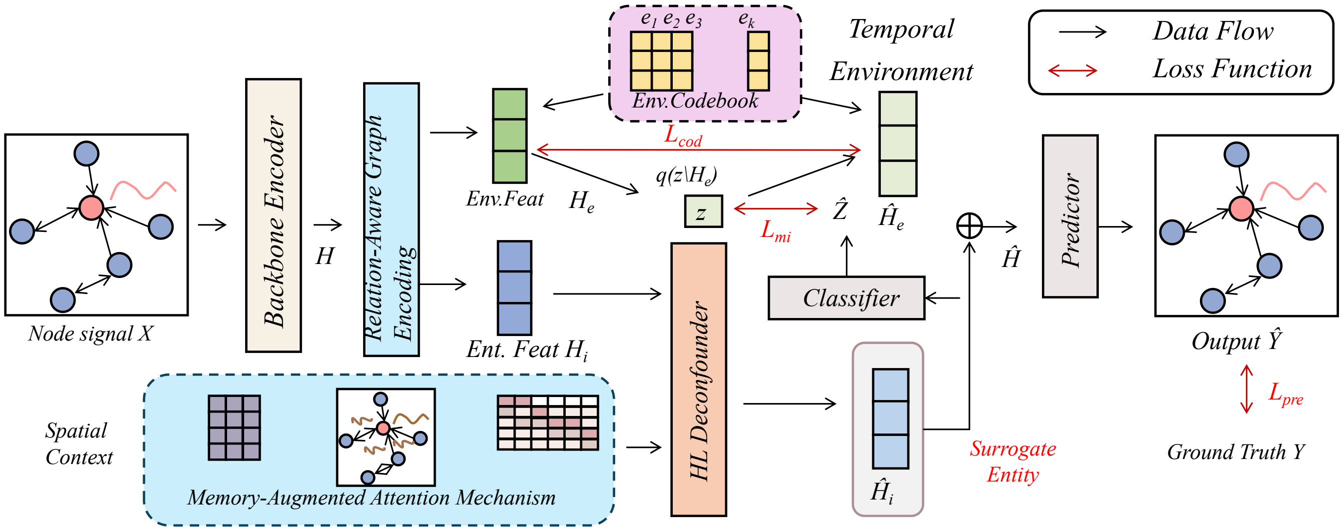

Figure S1. A schematic illustration of the Relational Pharmacological Embedding Network (PHARMNet) framework. The architecture integrates a backbone encoder and relation-aware graph encoding to process node signals and extract entity- and environment-specific features. A memory-augmented attention mechanism enhances spatial context representation, while a temporal environment module models domain-specific variability using a learned codebook. These components are further refined through HL deconfounding and classification, producing surrogate representations that are fused and passed to the predictor. Multiple loss functions—including prediction loss ($\mathcal{L}_{\text{pre}}$), latent mutual information loss ($\mathcal{L}_{\text{mi}}$), and environment coding loss ($\mathcal{L}_{\text{cod}}$)—jointly guide training to improve robustness and generalization across biomedical interaction domains.

Relation-Aware Graph Encoding.

Given a drug interaction graph $\mathcal{G} = (\mathcal{V}, \mathcal{E}, \mathcal{R})$ and node feature matrix $\mathbf{X} \in \mathbb{R}^{N \times F}$, we define an embedding layer,

$$\mathbf{H}^{(0)} = \mathbf{X}\mathbf{W}_0, \tag{S6}$$

where $\mathbf{W}_0 \in \mathbb{R}^{F \times d}$ is a trainable projection matrix, and d is the hidden embedding dimension(As shown in Figure S2).

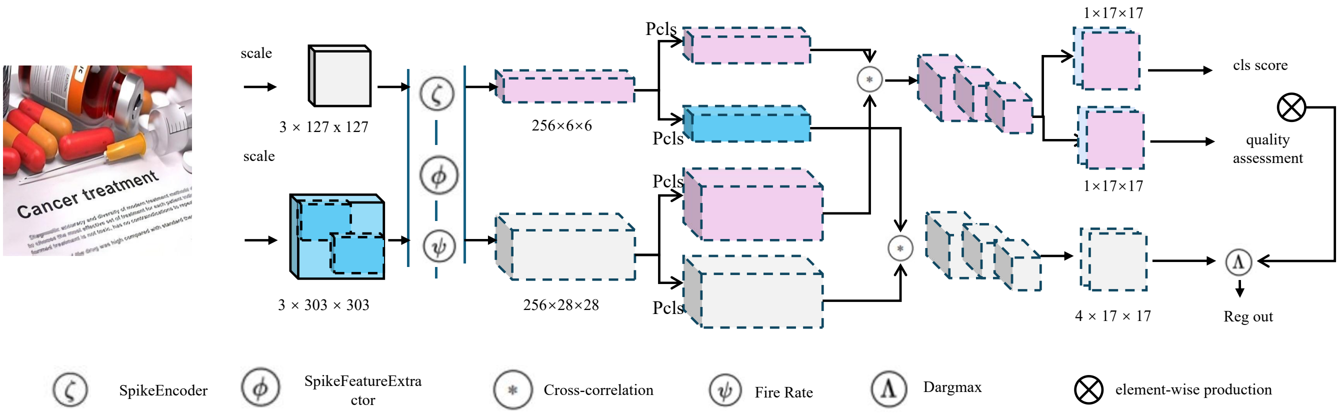

Figure S2. A schematic illustration of Relation-Aware Graph Encoding framework. The architecture includes dual-scale input modules processed through SpikeEncoder and SpikeFeatureExtractor blocks, followed by feature extraction at two resolutions, $256 \times 6 \times 6$ and $256 \times 28 \times 28$. These features undergo cross-correlation and element-wise multiplication to generate three parallel outputs, classification score maps ($1\times17\times17$), quality assessments, and regression outputs ($4\times17\times17$). Key computational components such as fire rate encoding, Dargmax selection, and weighted interaction pathways are integrated to enhance interpretability and multi-objective prediction across spatial contexts.

For each relation $r_k \in \mathcal{R}$, a relation-specific graph convolution is performed,

$$\mathbf{H}_k^{(1)} = \sigma\left(\hat{\mathbf{D}}_k^{-1}\hat{\mathbf{A}}^{(k)}\mathbf{H}^{(0)}\mathbf{W}_k\right), \tag{S7}$$

where $\hat{\mathbf{A}}^{(k)} = \mathbf{A}^{(k)} + \mathbf{I}$ is the adjacency matrix with self-loops for relation r_k, $\hat{\mathbf{D}}_k$ is the corresponding degree matrix, $\mathbf{W}_k \in \mathbb{R}^{d\times d}$ is a learnable weight matrix, and $\sigma(\cdot)$ is an activation function such as ReLU.

The relation-specific embeddings are aggregated via attention,

$$\mathbf{H}^{(1)} = \sum_{k=1}^{K} \alpha_k \mathbf{H}_k^{(1)}, \quad \text{with} \sum_{k=1}^{K} \alpha_k = 1, \tag{S8}$$

where the coefficients α_k are computed by a softmax-normalized attention over relation contexts,

$$\alpha_k = \frac{\exp\left(\mathbf{a}^\top \tanh(\mathbf{W}_a \bar{\mathbf{H}}_k)\right)}{\sum_{j=1}^{K} \exp\left(\mathbf{a}^\top \tanh(\mathbf{W}_a \bar{\mathbf{H}}_j)\right)}, \tag{S9}$$

with $\bar{\mathbf{H}}_k = \frac{1}{N}\sum_{i=1}^{N} \mathbf{H}_k^{(1)}[i,:]$, and $\mathbf{W}_a \in \mathbb{R}^{d\times d}$, $\mathbf{a} \in \mathbb{R}^d$ trainable.

Memory-Augmented Attention Mechanism.

To capture implicit drug interaction contexts and long-range dependencies not directly observable through graph structure alone, we introduce a learnable external memory bank $\mathcal{M} = \{\mathbf{m}_1, \ldots, \mathbf{m}_L\} \subset \mathbb{R}^d$ consisting of L memory slots. Each memory vector $\mathbf{m}_l$ serves as a latent prototype capturing common pharmacological motifs or interaction patterns observed during training. For each drug representation $\mathbf{h}_i$ derived from the relational encoder, we compute a soft attention distribution over memory slots via dot-product similarity,

$$\gamma_{il} = \frac{\exp(\mathbf{h}_i^\top \mathbf{m}_l/\sqrt{d})}{\sum_{j=1}^{L}\exp(\mathbf{h}_i^\top \mathbf{m}_j/\sqrt{d})}, \quad \forall i \in \{1,\ldots,N\}, \tag{S10}$$

where $\sqrt{d}$ is a temperature scaling factor to stabilize gradients and prevent over-sharpening. This produces a memory-aligned context vector via weighted aggregation,

$$\tilde{\mathbf{h}}_i = \sum_{l=1}^{L} \gamma_{il}\mathbf{m}_l, \tag{S11}$$

which acts as an auxiliary representation that complements the local structure-derived embedding $\mathbf{h}_i$. To enhance modeling flexibility, we introduce a gating mechanism that adaptively balances the influence of the original and memory-enhanced embeddings,

$$\mathbf{g}_i = \sigma\left(\mathbf{W}_g[\mathbf{h}_i \,\|\, \tilde{\mathbf{h}}_i] + \mathbf{b}_g\right), \quad \mathbf{g}_i \in [0,1]^d, \tag{S12}$$

where $\mathbf{W}_g \in \mathbb{R}^{2d\times d}$ and $\mathbf{b}_g \in \mathbb{R}^d$ are learnable parameters, and $\sigma(\cdot)$ denotes the sigmoid function. The final gated representation is computed as,

$$\mathbf{z}_i = \mathbf{g}_i \odot \mathbf{h}_i + (1-\mathbf{g}_i) \odot \tilde{\mathbf{h}}_i, \tag{S13}$$

where $\odot$ represents element-wise multiplication. To allow for further non-linear transformation and feature refinement, we pass $\mathbf{z}_i$ through a two-layer multilayer perceptron (MLP),

$$\mathbf{z}_i^{\text{final}} = \text{MLP}(\mathbf{z}_i) = \text{ReLU}(\mathbf{W}_2 \cdot \text{ReLU}(\mathbf{W}_1\mathbf{z}_i + \mathbf{b}_1) + \mathbf{b}_2), \tag{S14}$$

where $\mathbf{W}_1 \in \mathbb{R}^{d\times d}$, $\mathbf{W}_2 \in \mathbb{R}^{d\times d}$, and $\mathbf{b}_1, \mathbf{b}_2 \in \mathbb{R}^d$ are trainable parameters. This memory-augmented representation $\mathbf{z}_i^{\text{final}}$ captures both explicit relational information and latent pharmacological priors retrieved from the external memory, serving as a rich feature basis for downstream interaction prediction.

Prediction and Pharmacological Alignment.

For a given drug pair (d_i, d_j), the interaction prediction task is modeled as a multi-class classification problem over K possible interaction types. We first construct a composite interaction representation by combining the latent vectors $\mathbf{z}_i$ and $\mathbf{z}_j$ using element-wise multiplication and absolute difference,

$$\phi(\mathbf{z}_i, \mathbf{z}_j) = [\mathbf{z}_i \odot \mathbf{z}_j \,\|\, |\mathbf{z}_i - \mathbf{z}_j|], \tag{S15}$$

where $\odot$ denotes element-wise product and $\|$ indicates vector concatenation. This composite vector encodes both similarity and complementary patterns between the drugs. The interaction logits are then obtained via a linear transformation,

$$\hat{\mathbf{y}}_{ij} = \text{softmax}\left(\mathbf{W}_{\text{int}} \cdot \phi(\mathbf{z}_i, \mathbf{z}_j) + \mathbf{b}_{\text{int}}\right), \tag{S16}$$

where $\mathbf{W}_{\text{int}} \in \mathbb{R}^{K \times 2d}$ and $\mathbf{b}_{\text{int}} \in \mathbb{R}^{K}$ are trainable parameters, and the softmax function produces a probability distribution over K interaction classes. The supervised classification loss is defined using cross-entropy,

$$\mathcal{L}_{\text{int}} = -\sum_{(i,j)} \sum_{k=1}^{K} \mathbf{y}_{ij}^{(k)} \log \hat{\mathbf{y}}_{ij}^{(k)}, \tag{S17}$$

where $\mathbf{y}_{ij}^{(k)}$ is a one-hot encoded ground truth label. To inject domain knowledge and pharmacological consistency into the embedding space, we apply a target-level alignment loss. Let $\mathbf{Y}_i \in \mathbb{R}^{1 \times M}$ be the binary vector indicating known targets for drug d_i, and let $\mathbf{W}_{\text{bio}} \in \mathbb{R}^{M \times d}$ be a trainable projection matrix that maps target space into the embedding space. We enforce alignment between drug embeddings and their pharmacological target projections,

$$\mathcal{L}_{\text{bio}} = \sum_{i=1}^{N} \left\|\mathbf{z}_i - \mathbf{Y}_i \mathbf{W}_{\text{bio}}\right\|_2^2, \tag{S18}$$

which encourages $\mathbf{z}_i$ to be semantically grounded in molecular biology. To preserve smoothness over graph topology and promote consistency among neighboring drugs, we introduce a graph regularization loss. Let $\mathbf{A}$ be the adjacency matrix of the drug graph and $\mathcal{N}(i)$ denote the set of neighbors for drug d_i,

$$\mathcal{L}_{\text{reg}} = \sum_{i=1}^{N} \sum_{j \in \mathcal{N}(i)} \mathbf{A}_{ij} \left\|\mathbf{z}_i - \mathbf{z}_j\right\|_2^2, \tag{S19}$$

which penalizes large discrepancies between neighboring latent vectors. The final optimization objective combines the supervised prediction loss, graph-based regularization, and pharmacological alignment,

$$\mathcal{L}_{\text{total}} = \mathcal{L}_{\text{int}} + \lambda_{\text{reg}} \mathcal{L}_{\text{reg}} + \lambda_{\text{bio}} \mathcal{L}_{\text{bio}}, \tag{S20}$$

where λ_{reg} and λ_{bio} are hyperparameters that balance the contribution of each regularization term.

Interaction-aware Contextual Optimization with Pharmacological Evidence (INTERACT-SCOPE)

In this section, we present INTERACT-SCOPE, a knowledge-infused inference strategy that complements the PHARMNet architecture. This strategy targets the epistemic gaps in pharmacological data by dynamically refining interaction prediction based on contextualized priors derived from biomedical knowledge graphs, curated ontologies, and empirical pharmacokinetics(As shown in Figure S3).

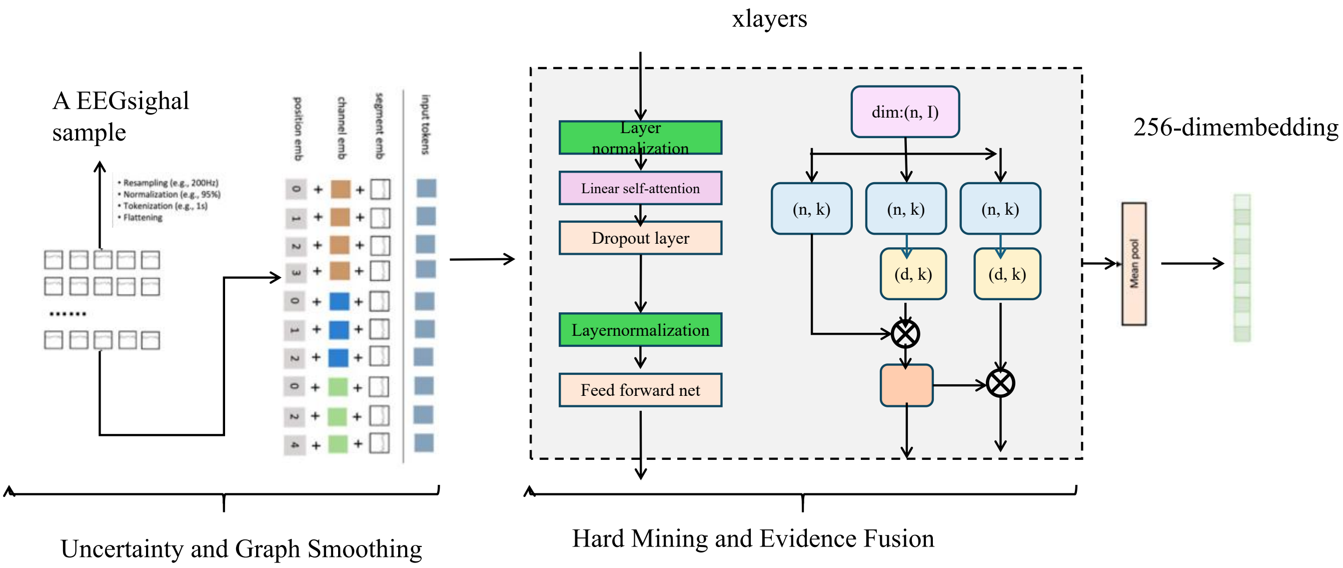

Figure S3. A schematic illustration of Interaction-aware Contextual Optimization with Pharmacological Evidence (INTERACT-SCOPE). The pipeline begins with preprocessing an EEG signal sample through steps including resampling, normalization, tokenization, and embedding of position, segment, and channel information. The processed input is passed through a transformer-style encoder composed of stacked layers with linear self-attention, layer normalization, dropout, and feedforward submodules. Within this structure, intermediate representations undergo interaction modeling and dimensional transformations, followed by element-wise operations and aggregation. A mean pooling operation produces the final 256-dimensional embedding, optimized through uncertainty modeling, graph smoothing, hard sample mining, and evidence fusion for robust neurophysiological feature extraction and classification.

Relation and Ontology Regularization.

Drug interaction networks are inherently sparse and exhibit strong label imbalance across interaction types, especially in multi-relational settings where certain pharmacological outcomes occur infrequently. This distributional skew introduces inductive bias and increases the risk of overfitting toward majority classes. To address these limitations, INTERACT-SCOPE incorporates structured domain knowledge by injecting relation-specific importance priors and ontology-based semantic consistency into the loss function(As shown in Figure S4).

Let $\mathbf{w} \in \mathbb{R}^K$ represent a learnable vector of relation importance weights. These weights are initialized using the inverse log-frequency of each relation type to counteract imbalance,

$$\mathrm{w}_k^{(0)} = \frac{1}{\log(1 + f_k)}, \quad f_k = |\{(i,j) : (d_i, d_j, r_k) \in \mathcal{E}\}|, \tag{S21}$$

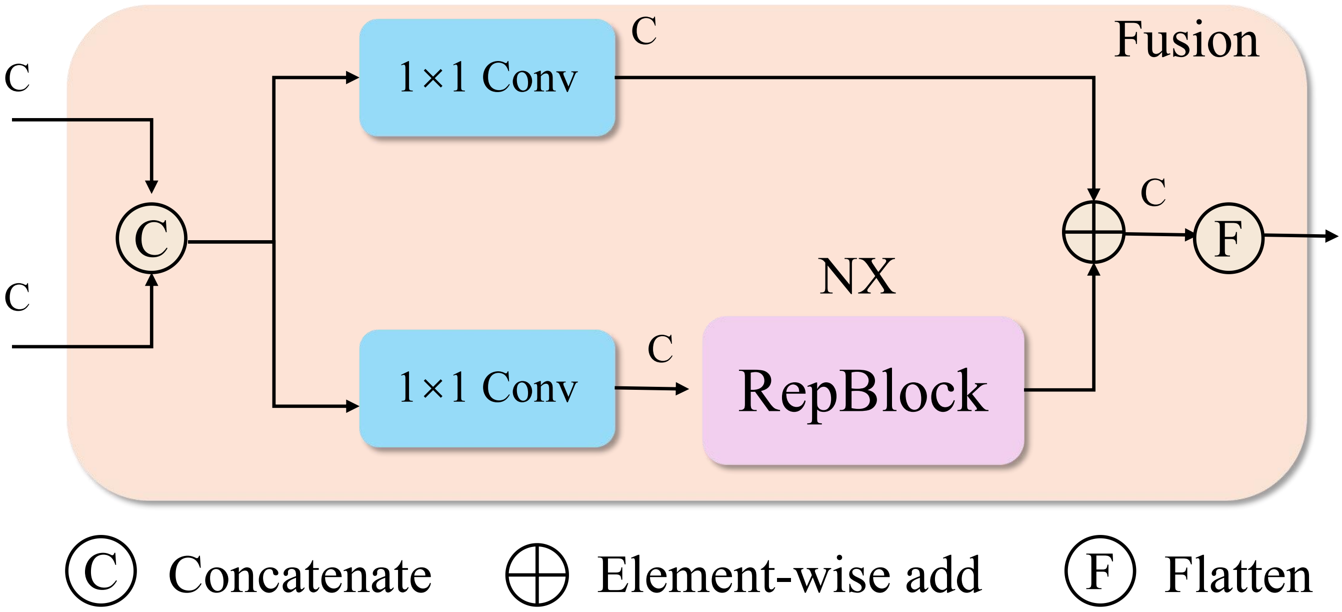

Figure S4. A schematic illustration of Relation and Ontology Regularization. The architecture processes input channels through parallel 1×1 convolution layers followed by a RepBlock component, which captures non-linear transformations. Inputs are first concatenated, then passed through the dual convolutional paths. The outputs are element-wise added and flattened to produce a unified feature vector. Symbols denote key operations, © for concatenation, $\oplus$ for element-wise addition, and F for flattening, forming a compact and expressive representation for downstream tasks.

where f_k is the empirical frequency of relation r_k in the training set. During training, these weights are adaptively updated via gradient descent to better reflect task-specific difficulty and informativeness. The weighted interaction loss becomes,

$$\mathcal{L}_{\text{int}} = \sum_{(i,j,k)\in\mathcal{E}} \mathbf{w}_k \cdot \ell(f_\theta(\mathbf{x}_i, \mathbf{x}_j)_k, \mathbf{y}_{ijk}), \tag{S22}$$

where $\ell(\cdot)$ is the standard cross-entropy loss and f_θ denotes the interaction predictor.

To impose structural consistency informed by biomedical ontologies, we define a regularization term that penalizes discrepancies in embedding space for drugs within the same therapeutic class. Let $\mathcal{O}$ be an external ontology encoding drug-taxonomy relationships. We construct a set $\mathcal{C}$ of drug pairs belonging to the same class,

$$\mathcal{C} = \{(i, j) \mid C(d_i) = C(d_j)\}, \tag{S23}$$

where $C(\cdot)$ retrieves the class label for a given drug. A consistency term encourages their latent embeddings to remain close,

$$\mathcal{L}_{\text{cons}} = \sum_{(i,j)\in\mathcal{C}} \|\mathbf{z}_i - \mathbf{z}_j\|_2^2 . \tag{S24}$$

To generalize this concept across hierarchical levels, we further weight each pairwise constraint by semantic similarity s_{ij} derived from path-based distances in the ontology,

$$s_{ij} = \frac{1}{1 + \text{depth}(\text{LCA}(d_i, d_j))}, \tag{S25}$$

where $\text{LCA}(d_i, d_j)$ is the lowest common ancestor of d_i and d_j in the ontology tree. The ontology-weighted consistency loss becomes,

$$\mathcal{L}_{\text{cons}}^{\text{ont}} = \sum_{(i,j)\in\mathcal{C}} s_{ij} \cdot \|\mathbf{z}_i - \mathbf{z}_j\|_2^2 . \tag{S26}$$

This loss encourages semantically close drugs to exhibit similar latent behavior, enhancing model generalizability across underrepresented pharmacological categories.

Uncertainty and Graph Smoothing.

We define epistemic uncertainty over the predicted interaction $\hat{\mathbf{y}}_{ij}$ using entropy,

$$\mathcal{H}_{ij} = -\sum_{k=1}^{K} \hat{y}_{ij}^{(k)} \log \hat{y}_{ij}^{(k)}, \tag{S27}$$

and introduce an uncertainty-weighted loss adjustment,

$$\mathcal{L}_{\text{unc}} = \sum_{(i,j)} \mathcal{H}_{ij} \cdot \|\hat{\mathbf{y}}_{ij} - \mathbf{y}_{ij}\|_2^2. \tag{S28}$$

We construct a similarity graph $\mathcal{S}$ over drugs based on target co-affinity, and define the Laplacian matrix $\mathbf{L} = \mathbf{D} - \mathbf{S}$, where,

$$\mathbf{S}_{ij} = \frac{|\mathcal{T}_i \cap \mathcal{T}_j|}{\sqrt{|\mathcal{T}_i| \cdot |\mathcal{T}_j|}}, \quad \mathbf{D}_{ii} = \sum_j \mathbf{S}_{ij}. \tag{S29}$$

We apply Laplacian regularization to the latent representations,

$$\mathcal{L}_{\text{lap}} = \text{tr}(\mathbf{Z}^\top \mathbf{L} \mathbf{Z}), \tag{S30}$$

where $\mathbf{Z} \in \mathbb{R}^{N\times d}$ stacks the latent vectors $\mathbf{z}_i$ row-wise.

Hard Mining and Evidence Fusion.

To enhance model discriminability under uncertain and ambiguous cases, we implement an adversarial hard mining strategy that focuses on samples with high prediction entropy and label disagreement from external knowledge bases. Let $\mathcal{H}_{\text{hard}}$ denote the set of hard samples defined as,

$$\mathcal{H}_{\text{hard}} = \left\{ (i,j) \mid \mathcal{H}_{ij} > \tau \wedge \arg\max_k \hat{y}_{ij}^{(k)} \neq \arg\max_k e_{ij}^{(k)} \right\}, \tag{S31}$$

where $\mathcal{H}_{ij}$ is the entropy of the prediction distribution $\hat{\mathbf{y}}_{ij}$, and $e_{ij}^{(k)}$ is the external prior score from databases such as DrugBank. For these challenging instances, we enforce sharper decision boundaries by employing a margin-based adversarial loss,

$$\mathcal{L}_{\text{adv}} = \sum_{(i,j)\in\mathcal{H}_{\text{hard}}} \max\left(0, \delta - \hat{y}_{ij}^{(k^+)} + \hat{y}_{ij}^{(k^-)}\right), \tag{S32}$$

where k^+ denotes the ground-truth interaction label, k^- is the highest scoring incorrect class, and δ is a positive margin hyperparameter ensuring separation. To better inform the learning process, we also assign adaptive weights to each hard instance based on its entropy,

$$\omega_{ij} = \frac{\mathcal{H}_{ij}}{\log K}, \tag{S33}$$

where K is the number of interaction classes. We scale the adversarial loss accordingly,

$$\mathcal{L}_{\text{adv}}^{\text{weighted}} = \sum_{(i,j)\in\mathcal{H}_{\text{hard}}} \omega_{ij} \cdot \max\left(0, \delta - \hat{y}_{ij}^{(k^+)} + \hat{y}_{ij}^{(k^-)}\right). \tag{S34}$$

This encourages the model to focus more on uncertain predictions while preserving margin-based decision confidence. The global optimization objective for INTERACT-SCOPE combines interaction loss, ontology alignment, uncertainty modeling, graph smoothing, and adversarial training,

$$\mathcal{L}_{\text{scope}} = \mathcal{L}_{\text{int}} + \lambda_1\mathcal{L}_{\text{cons}} + \lambda_2\mathcal{L}_{\text{unc}} + \lambda_3\mathcal{L}_{\text{lap}} + \lambda_4\mathcal{L}_{\text{adv}}^{\text{weighted}}, \tag{S35}$$

where $\lambda_1, \lambda_2, \lambda_3, \lambda_4$ are hyperparameters calibrated via grid search or Bayesian tuning.
